# Seroprevalence of SARS‐CoV‐2 in 10 Regional Capitals of Cameroon, October–December 2020

**DOI:** 10.1111/irv.13267

**Published:** 2024-03-27

**Authors:** Karampreet Sachathep, Yen T. Duong, Giles Reid, Emily Kainne Dokubo, Judith D. Shang, Clement B. Ndongmo, Ekali Gabriel, G. Tharp, Laura Eno Dimite, Adama N'Dir, Gordon Okpu, Francis M. Ogollah, Dubliss Nguafack, Martin C. Ntse, Gili Hrusa, Katherine Yuengling, Megan Tebbenhoff, Essomba René, Ngo Sack Françoise, Naah Tabala Felicity, Marie Claire Okomo, Anne‐Cecile Zoung‐Kanyi Bissek, Tiffany G. Harris

**Affiliations:** ^1^ ICAP at Columbia University New York New York USA; ^2^ Department of Population and Family Health Mailman School of Public Health, Columbia University New York USA; ^3^ US Centers for Disease Control and Prevention Atlanta Georgia USA; ^4^ US Centers for Disease Control and Prevention Yaoundé Cameroon; ^5^ ICAP in Cameroon Yaoundé Cameroon; ^6^ Department of Sociomedical Sciences Mailman School of Public Health New York New York USA; ^7^ Department of Epidemiology, Mailman School of Public Health Columbia University New York New York USA; ^8^ Ministry of Health Yaoundé Cameroon

**Keywords:** Cameroon, COVID‐19, SARS‐CoV‐2, serosurvey

## Abstract

**Background:**

Cameroon was among the most affected African countries during the first wave of the COVID‐19 pandemic; however, the true prevalence of SARS‐CoV‐2 remains unknown.

**Methods:**

From October to December 2020, we conducted a cross‐sectional, age‐stratified SARS‐CoV‐2 seroepidemiological survey at 30 purposively selected community‐based sites across Cameroon's 10 regional capitals, sampling 10,000 individuals aged 5 years or older. We employed a parallel SARS‐CoV‐2 antibody testing algorithm (WANTAI ELISA and Abbott Architect) to improve both the positive predictive value and negative predictive value of seroprevalence.

**Results:**

The overall weighted and adjusted seroprevalence of SARS‐CoV‐2 antibodies across the 10 urban capitals of Cameroon was 10.5% (95% CI: 9.1%–12.0%) among participants aged ≥5 years. Of the 9332 participants, 730 males (13.1%, 95% CI: 11.5%–14.9%) had SARS‐CoV‐2 antibodies compared to 293 females (8.0%, 95% CI: 6.8%—9.3%). Among those who reported a comorbidity at the time of testing, 15.8% (95% CI: 12.8%–19.4%) were seropositive. We estimated that over 2 million SARS‐CoV‐2 infections occurred in the 10 regional capitals of Cameroon between October and December 2020, compared to 21,160 cases officially reported at that time translating to one laboratory‐confirmed case being reported for every 110 SARS‐CoV‐2 infections across the 10 urban capitals.

**Conclusion:**

This study's findings point to extensive and under‐reported circulation of SARS‐CoV‐2 in Cameroon—an almost 100‐fold more cases compared to the number of cases reported to the World Health Organization. This finding highlights the importance of conducting serosurveys, especially in settings where access to testing may be limited and to repeat such surveys as part of pandemic tracking.

## 1 Introduction

Coronavirus disease (COVID‐19), caused by SARS‐CoV‐2, was first reported in China at the end of 2019. The highly contagious virus quickly reached pandemic proportions, and by the end of 2020, over 90 million COVID‐19 cases had been reported in 218 countries [1].

Cameroon had its first registered SARS‐CoV‐2 positive test in early March 2020 [2, 3]. Cases started to rise rapidly country‐wide, and by March 17, 2020, Cameroon was under a strict public health alert; wearing of masks became mandatory in all public areas, and education and awareness campaigns were implemented. Cameroon had the highest absolute number of cases in the central Africa subregion in 2020, with almost 30,000 reported cases and nearly 500 deaths [4]. However, the true number of SARS‐CoV‐2 infections in Cameroon is unknown because of both the likely large number of people with asymptomatic or mildly symptomatic infections and the lack of widespread testing [5]. Thus, determining the extent of community spread and establishing baseline seroprevalence data are needed to better understand population immunity levels and the impact of public health interventions, such as vaccinations, on future waves of transmission.

The purpose of this study was to estimate and describe the prevalence of SARS‐CoV‐2 antibodies among individuals aged ≥5 years in the 10 regional capitals of Cameroon. In addition, we aimed to determine factors associated with seropositivity. Finally, we gauge knowledge and attitudes towards COVID‐19. To the best of our knowledge, this was the first national SARS‐CoV‐2 serosurvey in Cameroon.

## 2 Methods

### 2.1 Survey Design and Population

We designed a cross‐sectional, age‐stratified SARS‐CoV‐2 seroepidemiological survey in community‐based sites across 10 regional capitals of Cameroon, in line with the World Health Organization (WHO) UNITY studies framework [6]. This survey took place from October to December 2020 at 30 purposively selected community‐based sites (three in each regional capital), including marketplaces, bus stops, and busy commercial intersections. High traffic venues were chosen because they most likely had a broad representation of age groups, sexes, and socioeconomic statuses. The sites were selected in conjunction with local stakeholders (Cameroon Ministry of Health, US Centers for Disease Control and Prevention [CDC] in Cameroon). For a more representative sample of the regional population, we set regional quotas for three broad age groups: 5–19 years, 20–49 years, and 50 + years (Appendix 1). The quotas were proportional to the regional population based on census data (2020) for each of these age groups [7].

Children <5 years were excluded due to the difficulty in obtaining blood from young children, especially in such public settings. At each site, survey staff used banners and other promotional materials and loudspeakers to announce the survey and attract attention to the on‐site survey tent. If participants were traveling in groups, only one adult and one child (5 years and above) per household were eligible to participate based on self‐reported relationships. Data were collected at each site for approximately 3 weeks, until quotas were met.

Prior to participation in the survey, written informed consent was obtained from adults (aged ≥21 years as per Cameroon regulations) and emancipated minors and from parents for participants aged ≤20 years. Written informed assent was also obtained from participants aged 10–20 years. The survey was approved by the Cameroon National Research Ethics Committee and Columbia University reviewed and designated it as public health surveillance. The study was reviewed in accordance with the US CDC human research protection procedures and was determined as research. However, the CDC investigators did not interact with any individuals or have access to identifiable data or specimens for research purposes.

#### 2.1.1 Procedures

Data were collected from October 19 to December 17, 2020, every day of the week with rotating recruitment teams. A questionnaire was first administered to participants (or their parent/guardian for those ages 5–14 years) using SurveyCTO on tablet. In this survey, which was adapted from the UNITY survey template, researchers collected information about sociodemographic factors; past COVID‐19 symptoms; potential COVID‐19 exposures (known contact with a laboratory‐confirmed case, travel [domestic or international], and health facility use); comorbid conditions and other risk factors; history of health‐seeking behavior; and knowledge, attitudes, and beliefs about COVID‐19. To asses past COVID‐19‐like illnesses, participants were asked whether, since the beginning of the COVID‐19 pandemic in January 2020 (before the first reported case in Cameroon), they had experienced any of the following symptoms per case definition used in Cameroon: dry cough, shortness of breath, tiredness, fever, muscle pain, diarrhea, headache, new loss of smell/taste, sore throat, nausea/vomiting.

After completing the questionnaire, 5 mL of venous blood was collected and placed in a cooler box with ice packs. After providing consent, participants who decided they did not want to complete the questionnaire were still allowed to provide their blood specimens. At the end of each day, samples were transported to a local laboratory where, using the plasma preparation tubes (PPTs), they were centrifuged to separate the plasma and then immediately frozen at −20°C. The frozen PPTs were transported under cold chain to the National Public Health Laboratory (LNSP) at the end of each week. At LNSP, the separated plasma in each PPT was pipetted into two 1 mL cryotubes (one cryotube for each assay) and frozen at minus −80°C until testing was performed.

Given the expected low seroprevalence (<10%) of SARS‐CoV‐2 antibodies in the Cameroon population at that time, a parallel testing algorithm was used to improve both the positive predictive value (PPV) and negative predictive value (NPV). Two enzyme‐linked immunosorbent assays (ELISA) tests available at the time (2020) were selected based on several factors: (1) sensitivity and specificity of available tests (minimum 95% sensitivity and 90% specificity, in line with US Food and Drug Administration Emergency Use Authorizations [US FDA EUA] validation requirements), (2) the best overall PPV and NPV of the testing algorithm using the FDA/CDC calculator on available tests on the market at that time point, (3) availability of plasma as the specimen type for testing, (4) authorization for use by US FDA EUA, WHO emergency use listing, CDC, or other internationally recognized bodies, and (5) test availability for purchase and shipment within a reasonable timeframe to complete this survey. The tests chosen were the Abbott Architect SARS‐CoV‐2 IgG (Abbott Diagnostics, Illinois, USA; 100% sensitivity and 99.6% specificity based on FDA report) and the WANTAI SARS‐CoV‐2 Ab ELISA (Beijing, China; 94.5% sensitivity and 100% specificity according to manufacturer's report). The WANTAI assay detects total antibody to the receptor binding domain (RBD) of the SARS‐CoV‐2 spike protein, including IgM, IgA, and IgG antibodies, while the Abbott assay only detects IgG antibodies against the nucleocapsid (N) protein. When used in combination, this testing algorithm theoretically should produce an overall PPV of ~100% based on a prevalence from 1% to 10% according to the FDA PPV calculator [8].

WANTAI ELISA testing was performed at the LNSP, while the Abbott Architect assay was performed at the Blood Bank of Yaoundé Central Hospital because of the availability of appropriate equipment. Each assay and test interpretation was performed according to the manufacturer's instructions and included appropriate quality controls to validate each run [9, 10]. After the testing was completed in the laboratory, remnant plasma specimens were returned to the −80°C freezers for long‐term storage.

A call center with set operational hours was established to return the results, which was listed on the consent forms provided to the participants. Survey participants would call and obtain their test results using a random identification number assigned at enrollment.

### 2.2 Statistical Analysis

**FIGURE 1:**
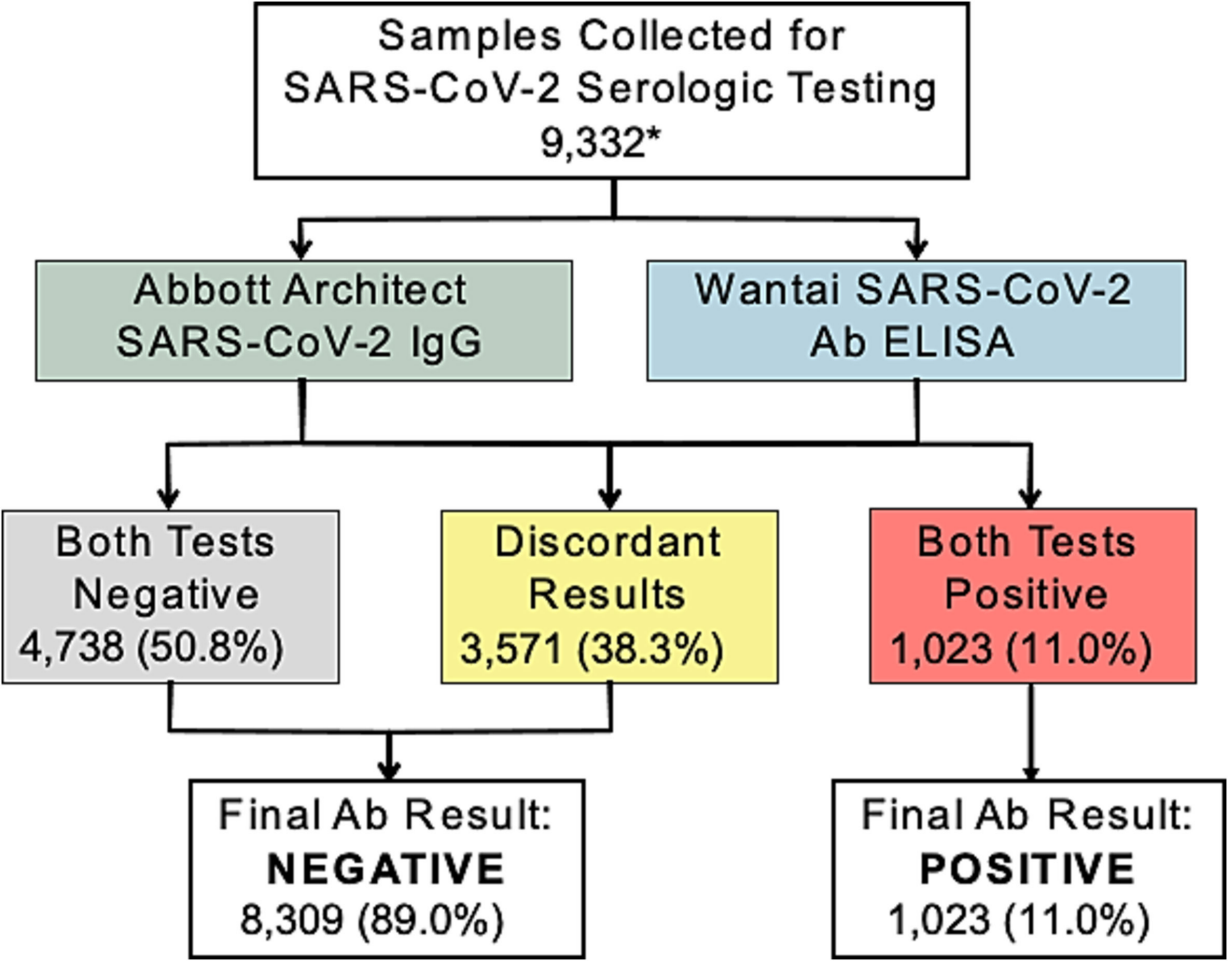
Testing outcome flowchart.

For the analysis, only those who tested positive on both WANTAI and Abbott assays were classified as SARS‐CoV‐2 antibody positive (Figure 1). Those who tested negative on only one or both assays were classified as negative.

Sampling weights were calculated based on selection probabilities at each site. Nonresponse weights (for questionnaire and each laboratory test) were calculated at the individual level. Weights were calibrated to regional population estimates by age and sex and then normalized to the total sample size [7]. Weighting was carried out in R using the glm, anesrake, and survey packages.

Seroprevalence estimation was done in SAS using the SURVEYFREQ procedure and the calibrated survey weights. Taylor series variance estimation was used to account for the clustered sample design, treating each survey site as a separate cluster. Unweighted counts and proportions were also computed in SAS. The χ^2^ test was used to assess the differences in prevalence across demographic characteristics.

We conducted unadjusted and multivariable logistic regression, incorporating survey weights, to assess the associations between demographic and behavioral characteristics with seropositivity. The analysis included participants with complete age and sex data. Final multivariable models included variables with *p* < 0.05 and variables (sex, age, residence, region, number of household members, comorbid conditions, and history of COVID‐19, travel) chosen *a priori* based on their likely association with the risk of SARS‐CoV‐2 past infection.

Regional‐level seroprevalence estimates were applied to 2020 region‐specific population projections from the Cameroon Statistics Agency to estimate the total number of SARS‐CoV‐2 infections in each region that had occurred in the survey period (October–December 2020). These numbers were compared with the total number of reported cases in the country at the end of the study (December 2020) to estimate the ratio of reported cases to total SARS‐CoV‐2 infections.

Agreement in test results between the WANTAI and Abbott assays was also evaluated using kappa statistics.

### 2.3 Results

Overall, 10,386 individuals were enrolled in the survey. Of these, 9180 (88.7%) completed an interview, 10,071 (97.0%) completed a blood draw, and 9686 (93.3% of 10,071) had results from both antibody assays. A total of 9332 (89.9% of 10,386) participants who had valid results from both assays as well as valid age and sex data were included in the analysis (Appendix 3). From both assays, 4738 (50.8%) participants tested negative, 1023 (10.9%) tested positive, and 3571 had discordant results resulting in a concordance of 61.7% and a kappa of 0.19 (Appendix 2). The WANTAI assay produced more positive results than the Abbott assay (4280 [45.8%] vs. 1337 [14.3%]).

Approximately half of the participants were female (51.2%, or 3548) and were aged 15–25 years (48.8% or 4529). All but one participant who enrolled was Cameroonian, and the majority had completed secondary education (43.1%) (Table 1). Only 9.2% of participants reported comorbid medical conditions (Table 1). Almost half of the participants age ≥15 years (43.7% or 3351 of 8399) reported being unemployed. The majority of those who were employed, worked in the informal trade sector (17.0%) (Table 1).

**TABLE 1:** Participant characteristics, October–December 2020 (*n* = 9332). Percent distribution of the study population by sex and selected demographic characteristics.

Characteristic	Total		
	*n*/*N* ^a^	Percent^b^	95% CI
Sex			
Male	5784/9332	48.8	47.5–50.1
Female	3548/9332	51.2	49.9–52.5
Age			
5–14	933/9332	7.6	7.0–8.2
15–25	4529/9332	48.4	47.1–49.8
26–35	1590/9332	18.7	17.6–19.8
36–45	902/9332	11.4	10.6–12.4
46–60	1051/9332	11.3	10.5–12.1
60 and above	327/9332	2.6	2.2–3.0
Residence			
Urban	8977/9332	96.8	96.3–97.2
Rural	351/9332	3.2	2.7–3.6
Missing	4/9332	0.0	0.0–0.1
Region of residence			
Far North	878/9332	11.0	10.3–11.7
North	1031/9332	7.2	6.7–7.8
Adamawa	750/9332	4.6	4.2–5.1
Center	894/9332	20.2	19.0–21.4
East	920/9332	5.9	5.5–6.4
South	999/9332	4.6	4.2–5.0
Littoral	893/9332	24.4	23.0–25.8
North West	936/9332	5.4	5.0–5.9
South West	1009/9332	4.5	4.1–4.9
West	981/9332	11.4	10.7–12.2
Missing	41/9332	0.9	0.6–1.2
Nationality			
Cameroonian	9331/9332	100.0	99.9–100.0
Other	1/9332	0.0	0.0–0.1
Educational attainment			
No education	449/9332	4.8	4.3–5.4
Primary	1318/9332	12.8	12.0–13.6
Secondary	3822/9332	41.5	40.2–42.8
Higher than secondary	3121/9332	37.3	36.0–38.6
Missing	622/9332	3.7	3.3–4.1
Self‐reported medical history^c^, ^d^			
Any history of comorbid medical condition	757/8399	9.2	8.4–10.1
Occupation^d^			
Healthcare worker	239/8399	3.1	2.6–3.7
Mining	8/8399	0.1	0.0–0.1
Agriculture/farming/fishing	253/8399	2.0	1.8–2.4
Transport	454/8399	6.0	5.4–6.7
Construction	196/8399	1.5	1.3–1.8
Uniformed personnel	148/8399	3.0	2.4–3.6
Informal trade	1389/8399	17.0	16.0–18.1
Garment industries	92/8399	1.0	0.8–1.3
Housekeeper	70/8399	1.3	1.0–1.8
Sex Worker	0/8399	0.0	0.0–0.0
Student	341/8399	2.7	2.3–3.0
Other employment	1536/8399	16.2	15.2–17.2
Unemployed	3351/8399	43.7	42.4–45.1
Missing/do not know/refused	322/8399	2.3	1.9–2.6

^a^ Denominators reflect the total number of participants with responses to the stratifier values listed.

^b^ Percentages in this table were calculated using survey weights.

^c^ Comorbid conditions surveyed about include hypertension, diabetes, heart disease, kidney disease, cancer, asthma, obesity, immunodeficiency, neurological disease, liver/lung disease, and other conditions.

^d^ Only collected for participants aged 15 years and older.

The overall weighted and adjusted seroprevalence of SARS‐CoV‐2 among those aged 5+ years across the 10 regional capitals of Cameroon was 10.5% (95% confidence interval [CI]: 9.1%–12.0%) among participants ≥5 years (Table 2). The seroprevalence was higher among males than it was among females (13.1% [95% CI: 11.5%–14.9%] vs. 8.0% [95% CI: 6.8%—9.3%]; *p* < 0.0001). Seroprevalence also differed by location (*p* = 0.0062), ranging from 7.5% (95% CI: 5.9%–9.5%) in the East region to 12.4% in the Far North (95% CI: 10.4%–14.8%) and North West (95% CI: 10.5–14.7) regions. Across age groups, seroprevalence ranged from 7.9% (95% CI: 5.9%–10.7%) among participants 5–14 years, to 19.2% (95% CI: 14.7%–24.8%) among those aged 60 years and above. Among those who reported a comorbidity at the time of testing, 15.8% (95% CI: 12.8%–19.4%) were seropositive. Further, among participants who were diabetic at the time of testing, 24.2% (95% CI: 15.4%–36.0%) tested positive for SARS‐CoV‐2 antibodies. Among participants who had reported recent travel, 15.9% (95% CI: 11.6%–21.5%) of those who had reported recent international travel were seropositive, and 11.9% (95% CI: 10.6%–13.4%) of participants who had reported recent domestic travel were seropositive (Table 2).

**TABLE 2:** Seroprevalence of SARs‐CoV‐2 and multivariable adjusted odds ratios by demographic and behavioral characteristics, October–December 2020.

Characteristic	*n*/*N* ^a^	Seroprevalence (%)^b^	95% CI	*p*‐value	Multivariable aOR (95% CI)^c^	Multivariable *p*‐value
Sex						
Male	730/5784	13.1	11.5–14.9	<0.001***	Ref.	<0.001***
Female	293/3548	8.0	6.8–9.3		0.61 (0.51, 0.74)	
Age						
5–14	80/933	7.9	5.9–10.7	<0.001***	Ref.	0.0125**
15–25	404/4529	8.2	6.9–9.7		1.01 (0.12, 8.75)	
26–35	178/1590	10.7	8.9–12.8		1.15 (0.12, 10.76)	
36–45	147/902	16.4	13.8–19.5		1.72 (0.21, 14.25)	
46–60	155/1051	13.4	10.5–17.0		1.46 (0.18, 11.53)	
60 and above	59/327	19.2	14.7–24.8		1.71 (0.17, 16.98)	
Total 5+	1023/9332	10.5	9.1–12.0			
Residence						
Urban	965/8977	10.2	8.9–11.8	0.0037**	Ref.	0.2438
Rural	57/351	17.2	12.0–24.0		1.32 (0.82, 2.11)	
Region of residence						
Far North	114/878	12.4	10.4–14.8	0.0062**	1.05 (0.72, 1.53)	<0.001***
North	92/1031	8.3	6.2–11.0		0.54 (0.30, 0.99)	
Adamawa	64/750	8.7	6.7–11.4		0.62 (0.41, 0.94)	
Center	110/894	11.8	8.7–15.8		0.93 (0.63, 1.39)	
East	80/920	7.5	5.9–9.5		0.57 (0.40, 0.82)	
South	121/999	11.7	9.6–14.1		0.86 (0.54, 1.36)	
Littoral	79/893	8.1	5.3–12.1		0.60 (0.40, 0.88)	
North West	119/936	12.4	10.5–14.7		1.01 (0.68, 1.51)	
South West	109/1009	11.8	8.1–16.7		Ref.	
West	129/981	12.3	10.4–14.5		1.03 (0.69, 1.52)	
Number of household members						
1–2	325/3024	10.2	8.6–12.2	0.6045	Ref.	0.6337
3–5	305/2639	10.8	8.9–13.2		1.12 (0.86, 1.46)	
6+	277/2274	11.5	9.2–14.2		1.11 (0.87, 1.41)	
Any comorbid condition						
Yes	111/678	15.8	12.8–19.4	<0.001***	1.18 (0.75, 1.87)	0.4656
No	689/6263	10.4	9.3–11.8		Ref.	
Diabetes						
Yes	21/83	24.2	15.4–36.0	<0.001***	1.63 (0.69, 3.84)	0.2561
No	779/6858	10.9	9.6–12.3		Ref.	
Hypertension						
Yes	38/250	16.4	12.0–22.0	0.0057**	1.10 (0.66, 1.84)	0.7031
No	762/6691	10.8	9.6–12.2		Ref.	
Ever tested SARS‐CoV‐2 positive						
Yes	8/27	(34.5) ^‡^	19.5–53.3	<0.001***	3.61 (2.01, 6.47)	<0.001***
No	1003/9190	10.4	9.1–12.0		Ref.	
Do not know	5/56	5.8	2.1–15.2		0.45 (0.09, 2.27)	
Travel within last week						
International	34/228	15.9	11.6–21.5	<0.001***	1.56 (1.10, 2.22)	0.035*
Domestic	462/3624	11.9	10.6–13.4		1.09 (0.92, 1.29)	
None	433/4299	9.5	8.1–11.2		Ref.	

*Note:* Seropositivity is defined as having a positive test in both antibody assays. The denominator includes all participants with valid results on both of these assays. Estimates based on a denominator less than 50 are shown in parentheses and should be interpreted with caution.

^a^ Denominators reflect the total number of participants with responses to the stratifier values listed.

^b^ Percentages in this table were calculated using survey weights.

^c^ Multivariable (adjusted) odds ratio estimates include participants with valid responses for all included stratifiers.

* *p* < 0.05,

** *p* < 0.01, and

*** *p* < 0.001.

In the multivariable analysis, females had a significantly lower seroprevalence than males (adjusted odds ratio [aOR]: 0.61, 95% CI: 0.51–0.74). Further, participants residing in the North (OR: 0.54 [95% CI: 0.30–0.99]), Adamawa (aOR: 0.62 [95% CI: 0.41–0.94]), the East (aOR: 0.57 [95% CI: 0.40–0.82]), and Littoral (aOR: 0.60 [95% CI: 0.40–0.88]) regions had a lower seroprevalence compared to those in the South West (Table 2). The odds of having tested positive for this virus were also significantly higher across older age groups (as compared to children ages 5–14 years) (aOR: 1.15 [95% CI: 0.12–10.76] for those ages 15–25 years and aOR: 1.72 [95% CI: 0.21–14.25] for persons aged 26–35 years). Seroprevalence was higher among participants who reported having ever tested SARS CoV‐2 positive (aOR: 3.61 [95% CI:2.01–6.47]) and among participants who traveled internationally in the past year compared to those who did not travel at all within the past year (aOR: 1.56 [95% CI: 1.10–2.22]). In multivariable analyses, seroprevalence did not differ by residential setting (urban vs. rural), history of comorbidities, or household size.

Based on the seroprevalence results from the study, we estimated that over 2 million persons were seropositive for SARS‐CoV‐2 (cumulatively) in the 10 regional capitals of Cameroon during October to December 2020, as compared to 21,160 cases officially reported at that time (Table 3) [11]. We estimate that one laboratory‐confirmed case was reported for every 110 SARS‐CoV‐2 infections across the 10 regional capitals. The highest number of SARS‐CoV‐2 infections was estimated to be in Center region with an estimated 475,000 infections, and the lowest number in Adamawa with an estimated 64,700 infections.

**TABLE 3:** Estimated number of SARS‐CoV‐2 antibody infections, age 5 years and above, October–December 2020.

	2020 population projection^a^	Estimated number of SARS‐CoV‐2 antibody infections
Region of residence		
Far North	1,102,255	137,200
North	3,868,987	320,900
Adamawa	739,186	64,700
Center	4,011,977	475,000
East	3,781,731	284,800
South	2,552,094	297,300
Littoral	1,999,153	161,800
North West	1,966,113	244,400
South West	668,334	78,700
West	1,740,333	213,500
Total	22,430,161	2,347,500

^a^ Population estimates derived from 2005 Census regional population distribution and UN population projections by age group. As of December 31, 2020, 26,277 COVID‐19 cases had been reported in Cameroon, out of 781,000 samples F tested (https://reliefweb.int/report/cameroon/cameroon‐covid‐19‐emergency‐situation‐report‐no‐13‐december‐2020).

## 3 Discussion

Overall, we found that an estimated 10.5% of Cameroonian survey participants aged ≥5 years were SARS‐CoV‐2 seropositive at the end of 2020. SARS‐CoV‐2 seroprevalence ranged widely by region (7.5%–12.4%) and seroprevalence was higher among males, persons older than 25 years of age, those who reported ever having tested positive for SARS‐CoV‐2 and among those who had reported having a recently traveled. However, seroprevalence did not differ by, residential setting (urban vs. rural), having a comorbid condition, or household size [12]. Another seroprevalence survey conducted in Cameroon that sampled households in Yaoundé, Cameroon, that was conducted around the same time as this study found a seroprevalence of 29.2% (95% CI 24·3–34·1) [13, 14]. This was more than 2.5 times higher than what we found for the seroprevalence (11.8%) in the Center region where Yaoundé is located. Like our survey, this survey also found that seroprevalence was higher among males. However, unlike our survey, they found a higher seroprevalence among participants living in larger households.

Our finding of 10.5% seroprevalence translates to an estimated 2,347,500 total infections across Cameroon at the time of the survey. This is over 100 times higher than the cumulative number of cases reported as of December 30, 2020 [11]. As of 2023, there were 124,392 cumulative confirmed cases and 1965 deaths reported in Cameroon due to SARs‐CoV‐2 [15]. A meta‐analysis that used data from seroprevalence studies conducted in 2020 found an overall seroprevalence of 19.5% in sub‐Saharan Africa, which varied widely and was significantly higher than that in high‐income countries [16]. The authors also found that seroprevalence estimates were a median 18.1 times higher than the cumulative incidence of reported cases overall and 600 times higher in sub‐Saharan Africa. Another meta‐analysis which used data published through December 2021 from Africa only (including data from our survey) also found a very high ratio (97:1) of seroprevalence to cumulative incidence that remained fairly constant over time [17]. Several reasons have been suggested for the seemingly low number of cases and even lower mortality rate and the misalignment between seroprevalence findings and reported cases in Africa [18, 19, 20]. These factors include lack of access to health services, including SARS‐CoV‐2 testing; limited public health surveillance capacity and infrastructure, including shortages of SARS‐CoV‐2 real‐time (RT)‐PCR test kits and other laboratory supplies; swift and wide‐reaching public health measures enacted by many countries; and the overall lower age of its population [18, 21]. Further, in 2020, the stigma associated with COVID‐19 along with misinformation and disinformation in the community likely resulted in testing avoidance, which exacerbated the undercounting of cases [22].

Even though our data are from the early period of the COVID‐19 pandemic, it and other studies highlight the importance of repeated seroprevalence surveys for providing a full picture of the extent of transmission in a country and to identify which groups are most affected. This has implications not only for ongoing surveillance of SARS‐CoV‐2 for which a substantial proportion of infections are asymptomatic or mildly symptomatic [23] but also for possible future pandemics caused by SARS‐CoV‐2 or other pathogens such as influenza that may not always cause symptoms. This is especially important when testing is low because of lack of availability or low demand or where a large proportion of the testing is conducted using at home tests that are not reported to health authorities. Repeated serosurveys are critical to understanding the epidemic both on a national and subnational level.

The prevalence estimates from this study were based on two assays: WANTAI SARS‐CoV‐2 Ab ELISA and Abbott Architect SARS‐CoV‐2 IgG. The WANTAI assay was made available by the WHO and was not independently evaluated at the time of the survey, while the Abbott assay was authorized for use by the US FDA after independently qualifying the assay with 100% sensitivity and 99.6% specificity [24]. In 2020, many antibody test kits entered the market with variable, and in some cases unreliable, test performance characteristics (sensitivity, specificity, PPV, and NPV) [25]. To overcome some of these test kit performance issues, we employed a parallel two‐test algorithm, as per FDA recommendations at the time, which increased the overall PPV for more accurate seroprevalence estimates. This approach differed substantially from that used by the majority of serosurveys conducted in the early days of the pandemic whereby a single test was employed to estimate prevalence, leading to uneven surveillance data quality [26, 27]. Some of these antibody tests included lateral flow immunoassays that had poorer performance than ELISA assays [28]. It is therefore very important to consider testing strategies when comparing different studies and that any interpretation of seroprevalence estimates should take into account individual assay performance characteristics, such as sensitivity and specificity.

In our parallel two‐test algorithm using the manufacturers' criteria for sample positivity, we found poor concordance between the WANTAI and Abbott assays (kappa value = 0.19), with the WANTAI assay producing a very high positivity rate of 45.9% compared to that of the Abbott assay, which was 14.3%. Similar to our findings, other studies have noted high positivity rates with the WANTAI assay compared to other ELISA assays [29, 30, 31]. One possible explanation for this discrepancy is that the WANTAI assay detects total antibodies to the receptor binding domain (RBD) of the SARS‐CoV‐2 spike protein, while the Abbott assay only detects IgG antibodies against the N protein. Assays that detect total antibodies have been shown to be more sensitive than those that detect either IgM or IgG and detect antibody earlier in infections (<21 days post‐symptom onset) [32, 33, 34]. Another possible contribution to the high positivity is the lower specificity due to cross‐reactivity to other circulating antibodies resulting from past infections from other pathogens. This has been noted in other evaluations, including WHO's own evaluation [35], resulting in false positives and higher overall prevalence estimates [36]. Conversely, the lower positivity rate from the Abbott assay may be due to the lower sensitivity associated with only detecting IgG antibodies and timing of testing from days post infection (<21 days). Given the shortcomings of both assays, as well as the unknown prevalence of COVID‐19 in Cameroon at the time of the survey, a parallel testing algorithm was used to reduce false positives and improve the overall PPV of the SARS‐CoV‐2 prevalence estimates for this survey. Using a criterion of positivity by either the WANTAI or Abbott assay would have resulted in a much higher seroprevalence of 49.2% (4594 positive samples/9332 total samples). This is likely to be an overestimation of true seroprevalence in this population, while using a two‐test serial algorithm may have resulted in an overall underestimation of seroprevalence due to the high specificity of the Abbott assay. The difference in testing strategies makes it difficult to compare these results head‐to‐head to other seroprevalence studies in Cameroon or elsewhere without adjusting for test performance characteristics. Rather, using the same testing strategy over time in the same study population can provide important information on changes in seroprevalence, either due to a public health intervention (i.e., vaccine campaign) or a new wave of infection.

This survey had several limitations. First, the use of convenience sampling from the regional capitals may indicate that the results are not representative of the entire population of Cameroon. However, the use of age stratified targets and post‐stratification with the participant weights was used to help reduce the effects of this selection bias. Second, bias may have been introduced because of experiences with COVID‐19, for example, people may have been more willing to participate if they or someone they know had been affected by COVID‐19, or they may have been less willing, if they felt like they had already contracted COVID‐19 and were not interested in finding out their antibody statuses. Third, the assays could have missed individuals who were still in the early stages of seroconversion. Our survey also has several strengths. It was the first SARS‐CoV‐2 seroprevalence survey that included all 10 regions of Cameroon and included over 10,000 adults and children aged ≥5 years. In each city, individuals were recruited from multiple sites to increase the diversity of participants. The survey also demonstrated the feasibility of performing a community‐based serological survey in large African urban centers. Further, we used two antibody tests to increase both PPV and NPV.

We conducted our survey in late 2020 before Cameroon experienced its second largest SARS‐CoV‐2 wave and two subsequent waves that likely increased seroprevalence [11], especially given that only 3% of the population had been vaccinated by the end of that year [37]. Repeated seroprevalence surveys after each large infection wave would have been useful to understand how readily the virus spreads in this population and also the impact of vaccines on the spread of the virus and any associated mortality.

## 4 Conclusion

Approximately one in 10 individuals in the regional capitals of Cameroon had been infected with SARS‐CoV‐2 by December 2020, indicating extensive and underreported circulation of SARS‐CoV‐2 in the country, with almost 100‐fold more cases across Cameroon as compared to the number of cases reported to the WHO. This finding highlights the importance of conducting seroprevalence surveys, which, if conducted using accurate testing algorithms, can capture a fuller burden of infection in a population, allowing public health officials to have more robust surveillance data and implement more effective responses and identify subgroups at higher risk for infection. With the availability of home testing and more mild disease, reported numbers of infections will become less reliable, and national seroprevalence surveys will become more important to understand disease burden and geographical impacts on a population.

## Author Contributions

*Study conception and design*: Karampreet Sachathep, Yen T. Duong, Giles Reid, Francis M. Ogollah, and Tiffany G. Harris. *Analysis and interpretation*: Karampreet Sachathep, Yen T. Duong, Giles Reid, Francis M. Ogollah, and Tiffany G. Harris. *Draft manuscript preparation*: Karampreet Sachathep, Yen T. Duong, Giles Reid, Emily Kainne Dokubo, Judith D. Shang, Clement B. Ndongmo, Ekali Gabriel, G. Tharp, Laura Eno Dimite, Adama N'Dir, Gordon Okpu, Francis M. Ogollah, Dubliss Nguafack, Martin C. Ntse, Gili Hrusa, Katherine Yuengling, Megan Tebbenhoff, Essomba René, Ngo Sack Françoise, Naah Tabala Felicity, Marie Claire Okomo, Anne‐Cecile Zoung‐Kanyi Bissek, and Tiffany G. Harris. All authors reviewed the results, contributed feedback and review, and approved of the final version of the manuscript.

## Conflicts of Interest

The authors have no conflict of interest to declare.

### Peer Review

The peer review history for this article is available at https://www.webofscience.com/api/gateway/wos/peer‐review/10.1111/irv.13267.

## Data Availability

The data that support the findings of this study are available from the corresponding author upon reasonable request.

## APPENDIX A

Sample Size Quotas by Age Group and Regional Capital, Cameroon 2020

**Table:**

Region (capital)	Age 5‐19 years	Age 20‐49 years	Age 50+ years
Far North (Maroua)	571	331	98
North (Garoua)	345	528	127
Adamawa (Ngaoundéré)	356	514	130
Center (Yaoundé)	524	350	126
East (Bertoua)	369	525	106
South (Ebolowa)	487	416	97
Littoral (Douala)	531	345	124
North West (Bamenda)	517	337	146
South West (Buea)	463	426	111
West (Bafoussam)	435	468	97
**Total**	4598	4240	1162

## APPENDIX B

Cameroon SARS‐CoV‐2 Serosurvey, Concordance of WANTAI SARS‐CoV‐2 Ab ELISA and Abbott Architect SARS‐CoV‐2 IgG Assay Results, October–December 2020

## APPENDIX C

Participant Flow Diagram
